# Supplementary figures and images for: Ultrastructural changes in cardiac and skeletal myoblasts following in vitro exposure to monensin, salinomycin, and lasalocid
Source: PLoS One. 2024 Sep 25;19(9):e0311046. doi: 10.1371/journal.pone.0311046 (PMC11423986; doi:10.1371/journal.pone.0311046)

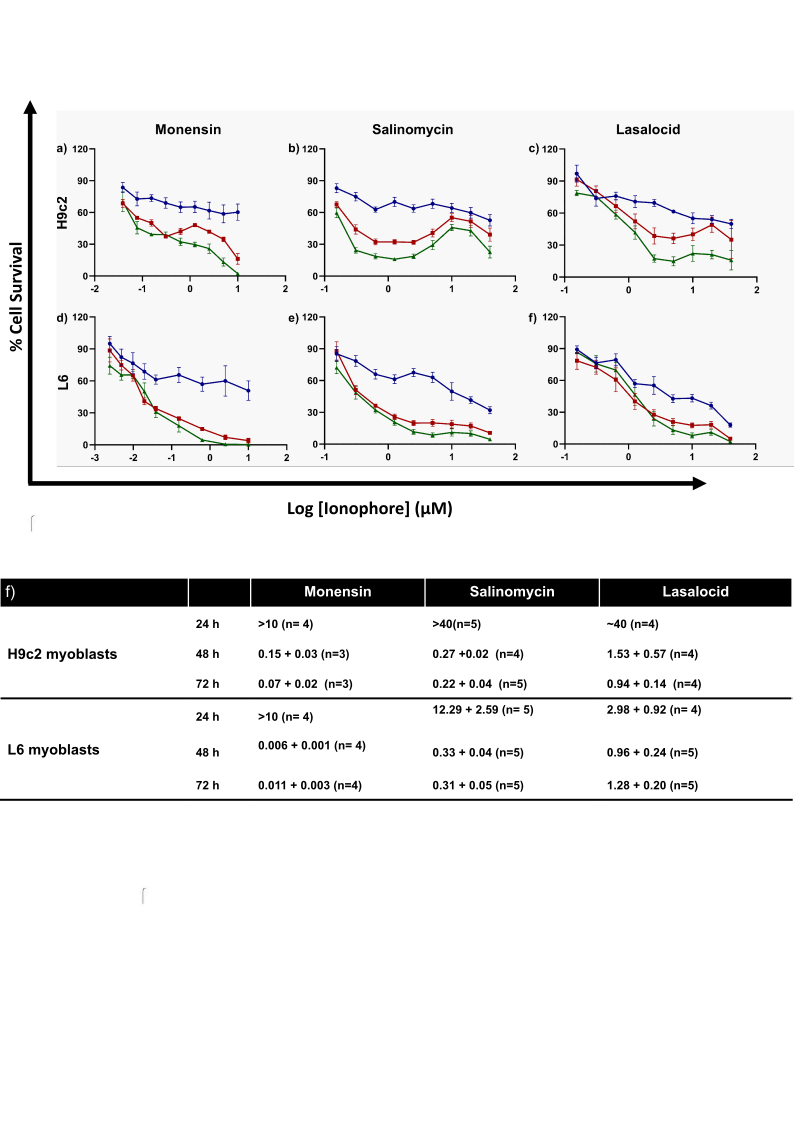

Supplement: S1 Fig — (a-f) Log-dose response curves were generated by using the mean percentage cell survival ± StEM vs the log of the concentration of the different ionophores (in μM). (Legend -●- 24 h, -■- 48 h, -▲- 72 h). (g) The EC50s (μM) ± StEM of the ionophores were exposed to three cell lines for 24, 48 and 72 h. n = number of biological repeats. (TIFF) [file pone.0311046.s001.tiff]

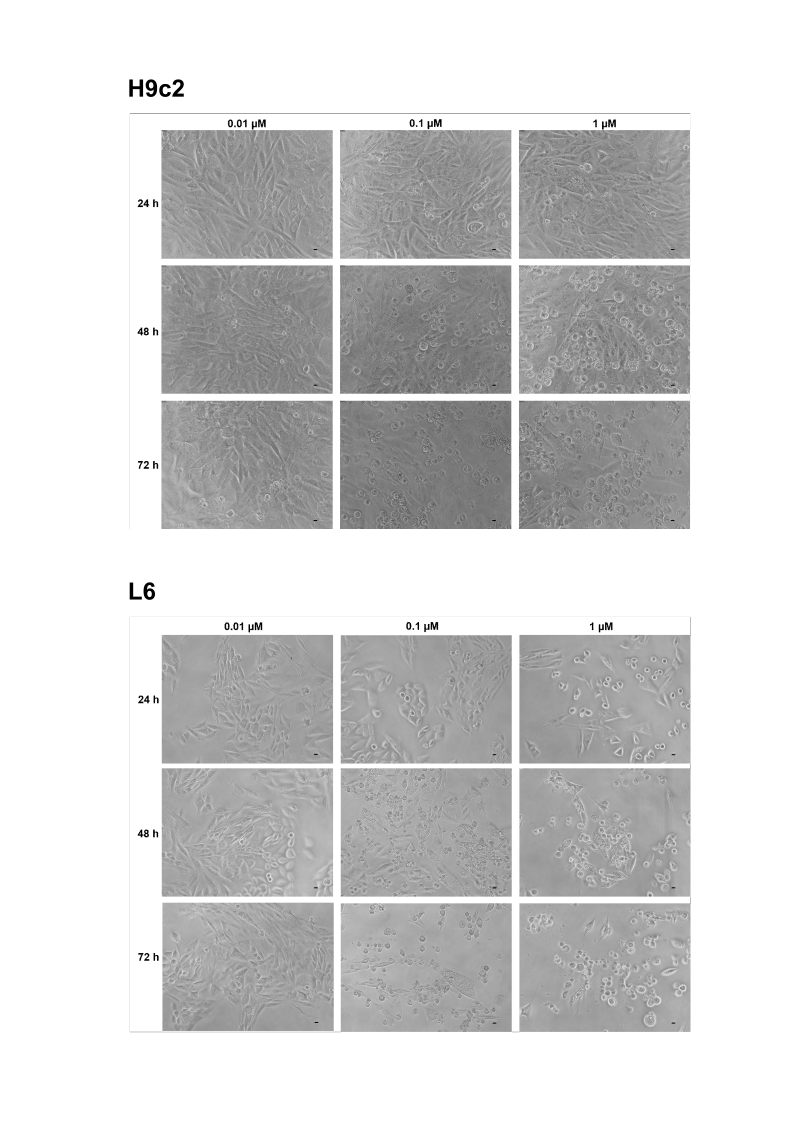

Supplement: S2 Fig — H9c2 and L6 myoblasts exposed to 0.01, 0.1 and 1 μM monensin. Scale bar = 10 μm. (TIFF) [file pone.0311046.s002.tiff]

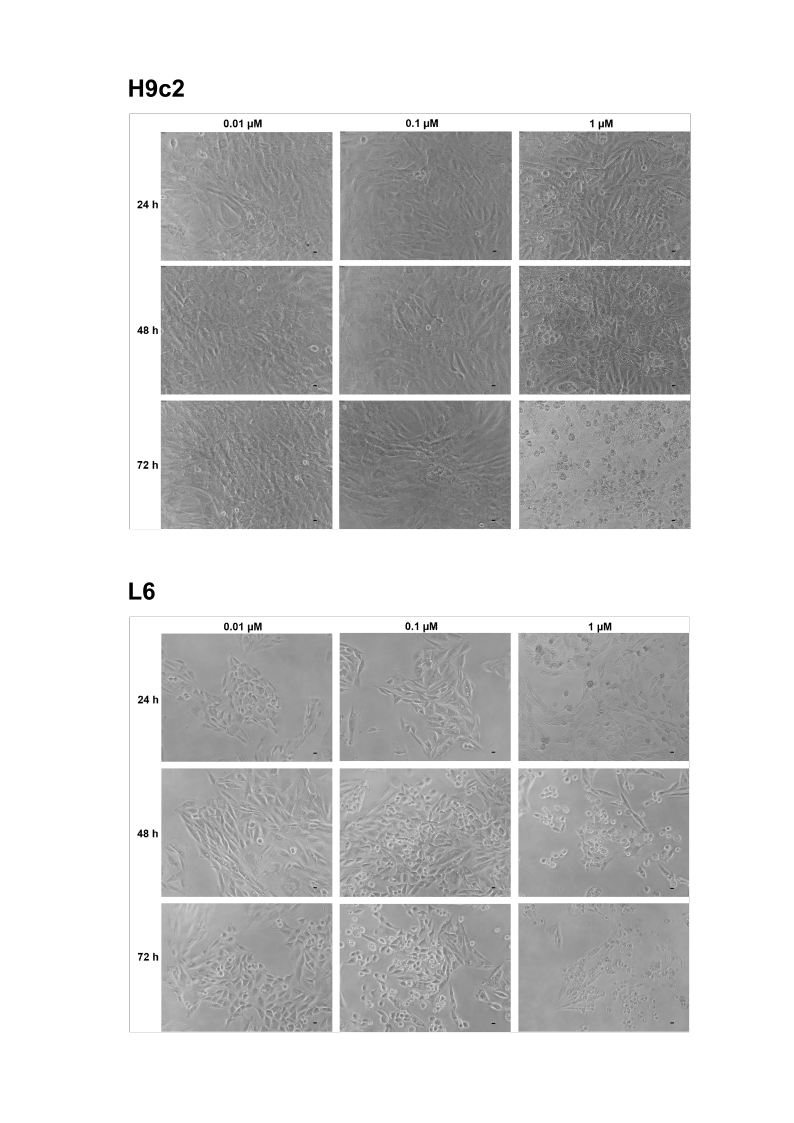

Supplement: S3 Fig — H9c2 and L6 myoblasts exposed to 0.01, 0.1 and 1 μM salinomycin. Scale bar = 10 μm. (TIFF) [file pone.0311046.s003.tiff]

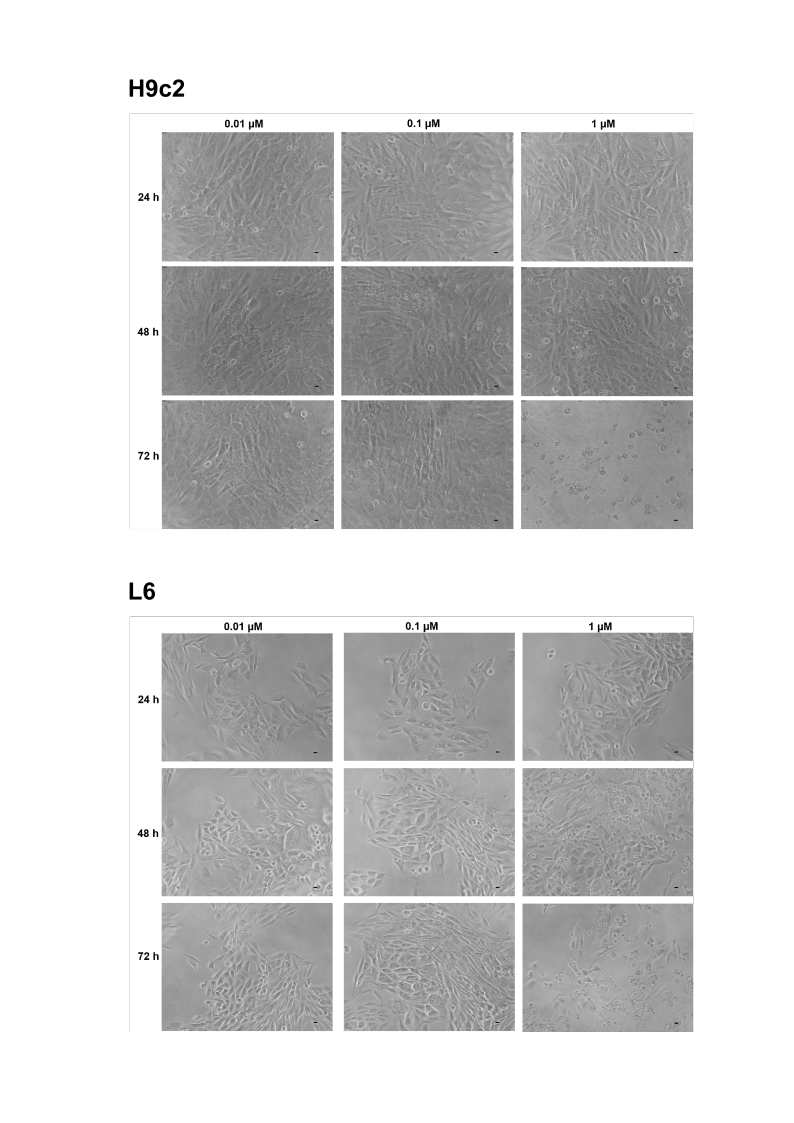

Supplement: S4 Fig — H9c2 and L6 myoblasts were exposed to 0.01, 0.1, and 1 μM lasalocid. Scale bar = 10 μm. (TIFF) [file pone.0311046.s004.tiff]
