# Supplementary material for: Ultrastructural changes in cardiac and skeletal myoblasts following in vitro exposure to monensin, salinomycin, and lasalocid
Source: PLoS One. 2024 Sep 25;19(9):e0311046. doi: 10.1371/journal.pone.0311046 (PMC11423986; doi:10.1371/journal.pone.0311046)
Supplement: Attachment — Submitted filename: Response to Reviewers.docx [file pone.0311046.s005.docx]

12 June 24

Dr Hans-Peter Kubis

**Academic Editor: PLOS ONE**

Dear Dr Kubis

Ms. Ref. No.: PONE-D-24-14459

Title: **Ultrastructural changes in cardiac and skeletal myoblasts following in vitro exposure to monensin, salinomycin, and lasalocid**

We are pleased to receive your communication on May 8, 2024, indicating a “Major revision” of our manuscript. The Editor and Reviewer’s comments were highly insightful and enabled us to improve the quality of the manuscript. I am pleased to submit the revised version of our manuscript.

We have revised the manuscript based on the editor and reviewer’s comments, observations, and suggestions to the best of our abilities.

We would like to thank you and the reviewers for your work in reviewing the manuscript and bringing up constructive inputs that will add value to the manuscript.

Our responses and answers to the editor and reviewer’s comments are hereby presented in red font below.

Please if there is any other thing that needs to be clarified, we will appreciate it if we can be given another opportunity to do so.

Kind regards,

**Dr AV Lensink
Manager, Electron Microscope Unit**

**Reviewer #1: Manuscript Number: PONE-D-24-14459**

In this manuscript submitted to Plos One journal as Research Article, the authors, with some experience in studying effects of ionophores in myocytes, reported interesting ultrastructural data obtained in vitro on two rat muscle cell lines (cardiac H9c2 and skeletal L6 myoblasts) consecutive to their incubation for 48 h with a single dose of three ionophore molecules, monensin, salinomycin, and lasalocid. The paper is concise and well written; the language is clear, unambiguous and in general correct (apart from several grammatical errors, or inadequate formulations, mentioned below). The paper has a good structure: Introduction provides enough background to illustrate the importance of the topic, Methodology is described in enough detail to allow replication of the experiments, the Results are relatively well presented (presentation must be improved), and the whole paper is based on classic references. The topic of the manuscript is relevant to the field of the Plos One journal, fits with the scope of this journal and could be of interest for certain readers of this journal. However, before recommending the publication of this manuscript, this reviewer considers that a major revision is required. Thus, there are 3 major and 32 minor aspects to be solved:

**Major points:**

1. The TEM and especially SEM images are of low quality and details are difficult to observe or are not visible at all in printed form. If the authors could not provide better images, they should ask the editing team (if paper will be accepted) whether is possible to improve somehow the images. Please replace Fig 5 d. A cell with disrupted plasma membrane is not representative for a control group. Magnification bars in SEM images, cannot be observed, and in all TEM and SEM images the corresponding dimensions (in µm) cannot be read. The authors could insert magnification bars in each panel of SEM images (according to their size in original images) and mention the corresponding dimensions in the legends of all panels in TEM/SEM figures.

**Thank you for bringing this to our attention. The figures were redone using different software which allows more precise control of exporting the images with specific size and resolution parameters. The PDF generated from the submission also significantly decreased the brightness and resolution – we will ask the editing team if it can be improved in the publication (if the paper is accepted).**

**Fig 5 d has been replaced with a more representative image for a control group sample.**

**New scale bars were added to the SEM images and scale bar dimensions were added to the legends of all panels.**

2. Apart from the aspects mentioned as “minor points” concerning the Discussion section, the authors have to compare in this section, and to discuss in more detail the results obtained for the two cell lines incubated with the three types of ionophores (and in relation with supplementary literature data). In this form of the manuscript, the authors only reported effects already known and mentioned into the scientific literature (by at least 9 papers cited here, and probably by many others that were not cited). Moreover, the authors have to point out the novelty of their approach in testing the toxicity of the three ionophores, the degree of originality of their results reported here, as well as the relevance of their original findings for the basic science and for the veterinary medicine (since not all the readers, including this reviewer are familiar with the problems raised by the cattle medication). They should also discuss the actuality of their work in a more general economic context (despite the fact that they did not use recent references – and if indeed there are no recent papers dealing with this topic, they should speculate the absence of such new data). Finally, the authors should discuss the extrapolation of their results to the clinical use of the tested ionophores.

**Thank you for the suggestions. We have elaborated on our discussion by making a more detailed comparison of the ultrastructural changes between the two cell lines, discussing previous findings as well as highlighting the novel factors of our approach. In our conclusion, we addressed the relevance of our data to basic/clinical research.**

**Of note, there is a general lack of recent literature published discussing ionophores (outside of cancer research).**

3. Please rewrite entirely the conclusions, based on the obtained results (instead of repeating some of the results), with possible recommendation for the future use of these ionophores in the veterinary medicine.

**As requested, we have rewritten the conclusion. Although this study does not focus on recommendations for future use of ionophores in veterinary medicine, it does contribute to our understanding of how pathological lesions develop in cardiac and muscle tissue following exposure to high ionophore concentrations.**

**Minor points (in the order of appearance):**

1. Lines 6 and 18: “¶These authors contributed equally to this work.” And is only one author labeled with this symbol.

**The superscript was changed to indicate the first two authors contributed equally.**

2. Line 30: in Abstract, please indicate the precise conditions of ionophores exposure (dose and time).

**The information on dose and exposure time was added.**

3. Line 39: Please be consistent with formatting of keywords.

**The keyword formatting was corrected.**

4. Line 51: Please be more specific since not all ionophores have the same molecular mechanism and the same effect on cells.

**We elaborated and specifically indicated carboxylic ionophores that form zwitterionic complexes, to distinguish them from neutral ionophores which form charged complexes and have a considerably greater toxic effect. We also clarified that the detrimental effect may occur, further mentioning in the next sentence that high levels of ionophores produce toxic effects.**

5. Lines 51-54: please distribute the citations more accurately, since is difficult to find the enumerated effects into the 10 references listed at the end of the paragraph.

**The citations were distributed to reflect where each cited effect was found in the literature.**

6. Lines 61-62. Please replace ”increased number of smooth endoplasmic reticulum and lipid droplets” with ”increased surface of smooth endoplasmic reticulum and number of lipid droplets”, since ER is a network of membrane within the cytoplasm. Alternatively, please use “number of ER profiles observed on the sections”.

**We replaced ”increased number of smooth endoplasmic reticulum and lipid droplets” with” increased surface of smooth endoplasmic reticulum and several lipid droplets”.**

7. Lines 89,90. If the authors cannot provide additional experimental data, please justify either here or in Discussion how the used dose of ionophores (0.1 µM) and the exposure time (48 h) were calculated (or selected) for incubation. For a better relevance of the result, at least two doses should be compared, or if a certain dose is more relevant, it should be tested for at least two different exposure times.

**Due to time and budget constraints, we had to limit our study to using only one concentration. Additionally, we have carried out in vitro cytotoxicity experiments to determine the EC50 of the different ionophores.** **The concentration of 0.1 uM was chosen as it was a concentration that was below the EC50 of both Lasalocid and Salinomycin, but higher than that for Monensin. Thus, it demonstrates the different degrees by which the ionophores can affect cardiac and skeletal myoblasts at the same concentration. We have added additional supplementary images in this regard.**

8. Line 135: “condensed mitochondria” are not visible in Fig. 2a-c. On the contrary, the presence of inner membranes and other debris in some of the electron lucent vacuoles is an indicative that mitochondria swelled and are responsible partially for the cytoplasmic vacuolation. This affirmation is supported by the round-shape of vesicles derived from mitochondria, while the expanded ER shows polymorphism. Moreover, this affirmation is also in line with the literature results cited in Introduction. Indeed, some condensed mitochondria are observed in Fig 2d, but this result is not mentioned in the main text.

**Thank you for the suggestion, this has been added and will improve the manuscript. The electron-lucent vacuoles are seen in Fig 2 a-c, and the condensed and swollen/fragmented mitochondria in Fig 2 b, the features have been labelled and referred to in the text.**

9. Line 136. It is very difficult to recognize the “autophagic vesicles” – not labelled in the panels a-c (and to delineate them from secondary lysosomes appearing in high number) in Fig. 2, since the double or multiple membranes of such vesicles could not be identified at this low resolution. The same comment for the other figures.

**The autophagic vesicles were labelled in Figures 2a and 2c and a higher magnification inset was added to Figure 2c.**

10. Line 140. Please explain here or discuss in Discussion the “indentations”, since they are not visible by TEM at the periphery of cells. This reviewer suggests that they resulted from the collapse of the multiple vacuoles either during dehydration followed by drying instead of epoxy infiltration, or/and during sputter-coating process, when the cells were exposed to a high vacuum.

**We agree with your suggestion and thank you for pointing this out. We have added this explanation.**

11. Line 144. The “apoptotic bodies” and “the red arrow” are not visible in Fig 2 g. It seems that authors inversed panels g and h of Fig 2 or explained inversely the results showed in the two panels.

**The image sequence and labels were corrected. Furthermore, we changed the image labels to be consistent throughout the manuscript (e.g. apoptotic bodies = circled, arrowheads = surface indentations, stars = condensed and marginalized chromatin, etc.).**

12. Please rephrase line 146.

**The sentence was rephrased and changed from ”Salinomycin had less of an effect on myoblasts than monensin” to “Salinomycin affected myoblasts to a lesser degree when compared with the monensin-exposed cells”.**

13. Line 148. Please indicate the panel(s) of Fig 3 showing the presence of autophagic vesicles and indicate these structures in the corresponding panel(s).

**The panel showing autophagic vesicles was referred to in the text and the corresponding panel was correctly labelled.**

14. There is no “pink arrow” in Fig 4h.

**Labels were added to Fig 4 h, please note that we discarded the pink arrows and consistently replaced the labels of all indicated surface indentations in the manuscript with arrowheads.**

15. Line 167. “A”, “G”, “M” and “RER” are not visible in the panels of Fig 2.

**Labels “A”, “M*” and “R” were added to the panels, no “G” was shown in this panel.**

16. Line 171. “A” and “RER” are not visible in the panels of Fig 3. In Fig 3c “G” should be placed in the lower left side of the image.

**The correct labels were added and G indicated on the lower left of Fig 3c**

17. Line 175. “pink arrows” and “A” are not visible in the panels of Fig 4.

**Labels were added to Fig 4 h, please note that we discarded the pink arrows and consistently replaced the labels of all indicated surface indentations in the manuscript with arrowheads. A – autophagic vesicles were added to Fig 4 a and c.**

18. Please rephrase line 180 (accord).

**The sentence was rephrased and changed from ”A large nucleus was generally located near the center of the myoblast, with one or more Golgi apparatus in close proximity” to “Myonuclei was typically centrally positioned, often with one or more adjacent Golgi apparatus visible”.**

19. Line 192. Please indicate the panels in Fig 6 showing “autophagic vesicles”.

**A label and the relevant in-text reference were added.**

20. Line 194. “Red asterisks” are not visible in Fig 6.

**The correct label was added to Figure 6c, please note that we discarded the red asterisk and consistently replaced the labels in all the figures showing condensed and marginalized chromatin with stars.**

21. Lines 190-196. Fig 6c is neither presented nor cited into the text.

**A reference to Fig 6c was added to the text.**

22. Lines 200-201. Fig 6g and 6h are inversely explained.

**The image sequence was corrected.**

23. Lines 202 and 205. The authors should indicate here or discuss later the origin of vesicles (numerous as seen in Fig 7a), since “mitochondria and RER were unaffected”.

**We have elaborated on the origin of the vesicles/vacuoles in the Discussion section**

24. Lines 206-208. Please check the correspondence of the presented results with the panels e-h of Fig 7.

**The image sequence was corrected, labels were added to the panels, and in-text references were corrected.**

25. Line 220. “A” is not visible in the panels of Fig 5.

**The correct label was added.**

26. Line 224. “red asterisks” are not visible in the panels of Fig 6. Please explain white asterisks.

**The label was added to Figure 6c, please note that we discarded the red asterisk and consistently replaced the labels of all the figures showing condensed and marginalized chromatin with stars. The white asterisk indicated the condensed mitochondria, however, this label was discarded and consistently replaced with “M*”, this was also added to the figure legend.**

27. Line 228. “A” is not visible in the panels of Fig 7.

**The correct label was added.**

28. Line 233. “The pink arrow” in Fig 8h is of the same colour as the “red arrow” in Fig 8d.

**The relevant labels were corrected, please note that we discarded the coloured arrows and consistently labelled all apoptotic bodies in the manuscript by circling them, and indicating the surface indentations with arrowheads.**

29. Line 234. “A” is not visible in the panels of Fig 8.

**The correct label was added.**

30. Please remove all references to figures from Discussion section.

**All the references to figures were removed from the Discussion section.**

31. Please rephrase line 289 (accord).

**The sentence was rephrased and changed from ”After ionophore exposure, only the Golgi apparatus of both H9c2 and L6 myoblasts were swollen, while the mitochondria were condensed, and the RER remained unaffected” to “After ionophore exposure, the Golgi apparatus in both H9c2 and L6 myoblasts displayed swelling, whereas the mitochondria appeared condensed, with no discernible effect observed on the rough endoplasmic reticula”.**

32. Line 301. Please replace “method” with “type” or “process”.

**As requested, we replaced “method” with “type”.**

**Reviewer #2: PLOS ONE Manuscript #: PONE-D-24-14459**

The authors investigated the effects of three carboxylic ionophores that are used as antibiotics in animal agriculture on ultrastructure of cardiac (H9c2) and skeletal muscle (L6) myoblasts using transmission and scanning electron microscopy. Ionophore exposure resulted in condensed mitochondria, dilated Golgi apparatus, and electron-lucent vesicles distributed throughout the cytoplasm. Apoptotic and necrotic myoblasts were observed after ionophore exposure. The compound Monensin had the largest effects on ultrastructure of cardiac and skeletal muscle myoblasts.

Although the manuscript provides interesting information on the effect of carboxylic ionophores on ultrastructure of cardiac and skeletal muscle myoblasts that deserve to be considered for publication in PlosOne, several issues need to be addressed.

**Major points**

Why do the authors restrict their analysis to myoblasts instead of investigating the effects of the ionophores also in myotubes?

**We agree that investigating the effects of the ionophores on myotubes would mimic the effects of the ionophores on actual muscle tissue better. Unfortunately, we had trouble consistently differentiating the myoblasts into myotubes. Budget and time constraints at the time of this study limited further optimization of the protocol.**

What is the rationale for using 0.1 μm for all three ionophores? Get treated animals actually the same dose of all three ionophores?

**Depending on the animal in question ionophores are usually administered as a feed additive in parts per million, which could be converted to mg/kg of the animal’s body weight, depending on the rate of ingestion of the feed. There is a large variation in toxic dose or lethal dose (LD50) between species (i.e. interspecies differences) with e.g. horses approximately 10 times more susceptible than cattle with regard to monensis toxicosis. The toxic dose of the different ionophores also differs within species. We have previously carried out in vitro cytotoxicity experiments to determine the EC50 of the different ionophores. The concentration of 0.1 uM was chosen as it was a concentration that was below the EC50 of both Lasalocid and Salinomycin, but higher than that for Monensin. Thus, it demonstrates the different degrees by which the ionophores can affect cardiac and skeletal myoblasts at the same concentration.**

An Ethics Statement is missing in the Methods section.

**An ethics statement has been added to the Methods section.**

Lines 300-302: Concerning distinction of ionophore-induced apoptosis and necrosis, the authors state that “morphological criteria alone is not sufficient”. In accordance, to assess myoblast apoptosis, annexin V staining should be considered to evaluate effects of ionophore treatment. Levels of lactate dehydrogenase (LDH), a marker of necrosis, should be determined in the supernatant of ionophore-treated myoblasts.

**We agree with the comment, but it was not the objective of this electron microscopy study. However, we have added the suggestion of the reviewer to highlight that additional tests are required to distinguish between cell death modalities.**

Several mismatches and inconsistencies between Results, Figures and Figure Legends need to be revised thoroughly:

Lines 135-136: “Condensed mitochondria (Fig 2 a-c)” are shown in Figure 2d.

**The image sequence, labels, and in-text references have been corrected.**

Lines 136, 148, 155-156, and 192-193: How are “autophagic vesicles“ defined ultrastructurally and distinguished from other vesicles? Please indicate them in Figures 2a-c, 3a-c, 4, and Figure 6. Concerning autophagic vesicles, see also comments on Figure Legends below.

**An ultrastructural description/definition has been added to the first mention of autophagic vesicles (H9c2 myoblast monensin exposure results) and labels to indicate these vesicles added to Figures 2a & c, 3a-c, 4a & c.**

Lines 138, 143, 170, 196, 224, and 232: Which features define the budding structures as “apoptotic bodies“?

**An ultrastructural description/definition has been added to the first mention of “apoptotic bodies” (H9c2 myoblast monensin exposure results).**

Lines 138-139, line 144: Please indicate features of necrosis like cellular debris in Figures 2d and h.

**A description of the loss of membrane integrity and cytoplasmic degradation has been added and the features indicated in Figures 2 d and h.**

Lines 143-144: A red arrow supposed to mark apoptotic bodies in Figure 2g is shown in Figure 2h.

**The image sequence and relevant labels were corrected, please note that we discarded the coloured arrows and consistently labelled all apoptotic bodies in the manuscript by circling them.**

Line 160. The pink arrow supposed to mark indentations is missing in Figure 4h.

**The relevant labels were corrected, please note that we discarded the coloured arrows and consistently labelled the surface indentations in all the relevant figures with arrowheads.**

Legends Figures 1 to 4: several features listed in the Legends are not marked in the corresponding Figures. Figure 1: RER; Figures 2 and 3: A-autophagic vesicles, RER; Figure 4: A-autophagic vesicles.

**The relevant labels were added to the figures.**

Line 194. What means “marginalized chromatin? White but not red asterisks (see line 224) are shown in Figure 6b and d, obviously indicating mitochondria but not “condensed chromatin” (line 223-224).

**A description/definition of “marginalized” was added to the text, and the labels were corrected on the relevant figures. Please note that we discarded the red asterisk and consistently replaced the labels of the condensed and marginalized chromatin in the manuscript with stars. The white asterisk indicating the condensed mitochondria was also discarded and consistently replaced with “M*”.**

Line 195. Please indicate “condensed cytoplasm” in Figure 6.

**A label indicating the condensed cytoplasm was added and described in the figure legend.**

Line 197-198. Please rephrase: “…resulted in the surface of many myoblasts containing deep indentations scattered around the nucleus…”.

**The sentence was rephrased and changed from ”Monensin exposure, similar to H9c2 myoblasts, resulted in the surface of many myoblasts containing deep indentations scattered around the nucleus, giving the myoblasts a pockmarked appearance” to “Monensin exposure, similar to what was seen in the H9c2 myoblasts, resulted in deep indentations on the surface of many myoblasts, giving the myoblasts a pockmarked appearance”.**

Line 200: Please specify “lacked surface structures”.

**The surface structures were elaborated on in the control group result section, and the lack of surface structures (concerning what was seen in the control group) has been described in the monensin exposure result section.**

Line 201: Please indicate features of necrosis in Figure 6h.

**A typical feature of necrosis (loss of membrane integrity) has been indicated in Figure 6h.**

Legends Figures 5 to 8: several features listed in the Legends are not marked in the corresponding Figures. Figure 6: A-autophagic vesicles, RER, red asterisks supposed to mark condensed (line 223-224) chromatin are white and indeed mark mitochondria, and h is supposed to show necrotic myoblasts (line 201), while the red arrow is supposed to show apoptotic bodies (line 224); Figure 7: A-autophagic vesicles; Figure 8: A-autophagic vesicles, a pink arrow supposed to show indentations (lines 214-215) is not shown in Figure 8g.

**All the relevant features listed in the figure legends (and in-text) have been added and the image sequence corrected. Please note, we changed the figure labels to be consistent throughout the manuscript (e.g. condensed chromatin = stars, condensed mitochondria = M*, apoptotic bodies = circled, surface indentations = arrowheads).**

Please provide a conclusion based on your observations.

**As requested, we have rewritten our conclusion.**

**Minor points**

Lines 130-133, and lines 187-189. These summaries should preferably appear at the end of respective paragraphs.

**The two summaries have been moved to the end of each respective paragraph.**

Lines 55-58: Concerning ionophore toxicosis, the authors argue about “a variable inflammatory component”. What are the ultrastructural features of inflammation? Are signs of inflammation observed in the myoblasts?

**The inflammatory response referred to is a systemic response that has been seen in vivo as lymphocyte and macrophage infiltration. In the current study, we do not expect to see signs or ultrastructural features of inflammation. However, the ultrastructural changes observed (for example necrosis) could be endogenous triggers of inflammation that influence the pathological outcome of toxicosis *in vivo*.**

Lines 243-244: “Monensin blocks transport at the trans-face of the Golgi apparatus….” Is this statement a conclusion from the data? Otherwise, please provide a reference.

**Thank you for bringing the lack of reference to our attention, the relevant reference has been added.**

Lines 248-249: What is the evidence that electron-lucent vesicles filling the entire myoblasts are the result of dilatation of the Golgi apparatus?

In this context:

What is the evidence for statements relating intracellular vesicles “distributed throughout the cytoplasm” (line 31) to indentations? Lines 31-32: “…electron-lucent vesicles distributed throughout the cytoplasm, which appeared as indentations on the myoblast surface.” Similar: Lines 240-241: “…accumulation of electron-lucent vesicles within the cytoplasm of the affected myoblasts, which manifested as indentations… “. Lines 249-250: “Vesiculation of the cytoplasm results in indentations on the myoblast surface, giving the myoblasts a pockmarked appearance.” Lines 304-305: “The vesicles manifest as indentations on the surface of the myoblasts.”

**We did not mean to infer that the electron-lucent vesicles are solely the result of the dilation of the Golgi apparatus, as ultrastructural evaluation alone cannot confirm this. The relevant sentences have been rephrased to mention the Golgi dilation and vacuolization as separate observations to remove the ambiguity.**
**We believe the indentations observed with SEM are a result of the collapse of the vacuoles during drying – we added this to the first mention of the observed feature in the result section.**

Lines 283-284: Please rephrase and specify “surface details”.

**An elaborated description of the surface details has been added, and the term rephrased throughout the manuscript.**

Lines 277-279, and 287-288. The characteristic morphological features of apoptosis and necrosis should be introduced in the Introduction. Please provide a reference for apoptotic features.

**A section describing the characteristic features of apoptosis and necrosis with references has been added to the introduction.**

Line 295: Please rephrase “…. can drive a cell to favor necrosis over apoptosis.”

**The sentence has been rephrased and changed from ”Calcium overload and reduced mitochondrial respiration, with the consequent lack of cellular energy, can drive a cell to favor necrosis over apoptosis” to “Calcium overload and reduced mitochondrial respiration, with the consequent lack of cellular energy and cell injury, can trigger necrotic cell death mechanisms over more conserved apoptotic pathways”.**
