# Supplementary material for: Ultrastructural changes in cardiac and skeletal myoblasts following in vitro exposure to monensin, salinomycin, and lasalocid
Source: PLoS One. 2024 Sep 25;19(9):e0311046. doi: 10.1371/journal.pone.0311046 (PMC11423986; doi:10.1371/journal.pone.0311046)
Supplement: Attachment — Submitted filename: Response to Reviewers.docx [file pone.0311046.s006.docx]

19 August 2024

Dr Hans-Peter Kubis

**Academic Editor: PLOS ONE**

Dear Dr Kubis

Ms. Ref. No.: PONE-D-24-14459

Title: **Ultrastructural changes in cardiac and skeletal myoblasts following in vitro exposure to monensin, salinomycin, and lasalocid**

We are pleased to receive your communication on July 5, 2024, indicating a “Revision required” of our manuscript. The Editor and Reviewer comments enabled us to improve the quality of the manuscript. I am pleased to submit the revised version of our manuscript. We have revised the manuscript based on the editor and reviewer’s comments, observations, and suggestions to the best of our abilities.

We would like to thank you and the reviewer for your work in reviewing the manuscript and bringing up constructive inputs that will add value to the manuscript.

Our responses and answers to the editor and reviewer’s comments are hereby presented in red font below.

Please feel free to let us know if there is any other thing that needs to be clarified, we will happily do so and appreciate it if we are given another opportunity to do so.

Kind regards,

**Dr AV Lensink
Manager, Electron Microscope Unit**

**Reviewer #2: PLOS ONE Manuscript #: PONE-D-24-14459R1
Title: Ultrastructural changes in cardiac and skeletal myoblasts following in vitro exposure to monensin, salinomycin, and lasalocid.
Authors: Danielle Henn; Antonia Vergina Lensink; Christo J Botha**

The authors investigated the effects of three carboxylic ionophores that are used as antibiotics in animal agriculture on ultrastructure of cardiac (H9c2) and skeletal muscle (L6) myoblasts using transmission and scanning electron microscopy. Ionophore exposure resulted in condensed mitochondria, dilated Golgi apparatus, and electron-lucent vesicles distributed throughout the cytoplasm. Apoptotic and necrotic myoblasts were observed after ionophore exposure. The compound Monensin had the largest effects on ultrastructure of cardiac and skeletal muscle myoblasts.
Although the revised manuscript is significantly improved in response to the reviewer’s comments, again several issues especially related to Results, Figures and Figure Legends need to be addressed.

**Major points**

Line 167 [Numbers from manuscript with Track Changes]: In Figure 2b, an inset supposed to demonstrate “mitophagy-like structures” is missing.

**Thank you for pointing out this mistake, the correct figure has been uploaded.**

Line 168: What does “mitochondrial vacuolar degeneration” mean? The structures shown in Figure 2b related to category M° (lines 222-223), supposed to show “mitochondrial vacuolar degeneration”, are not convincing.

**Mitochondrial vacuolar degeneration has been described as the derangement of mitochondrial structural integrity which can be morphologically characterised by progressive stages. Initially the mitochondrial cristae undergo conformational changes, followed by focal loss and dissolution of the cristae. Advancing stages show the multifocal loss of the cristae, mitochondrial swelling, significant to complete dissolution of the cristae, permeabilization and loss of the inner mitochondrial membrane and eventually the rupture of the outer mitochondrial membrane. This is a common aetiology of oxidative stress, ROS production and increased mitochondrial calcium which also results from toxic ionophore exposure.**

**Upon the recommendation of another reviewer: “**…presence of inner membranes and other debris in some of the electron lucent vacuoles is an indicative that mitochondria swelled and are responsible partially for the cytoplasmic vacuolation. This affirmation is supported by the round-shape of vesicles derived from mitochondria…”, **and further comparison of micrographs depicting mitochondria undergoing vacuolar degeneration in literature (references 1- 5 below) we decided to include this terminology to highlight the degree of mitochondrial damage observed. However, we have rewritten the relevant section, adding a further morphological description and changing the word choices to convey the fact that these structures is suggestive of mitochondrial vacuolar degeneration.**

1. Chaanine AH. Morphological Stages of Mitochondrial Vacuolar Degeneration in Phenylephrine-Stressed Cardiac Myocytes and in Animal Models and Human Heart Failure. Medicina (Kaunas). 2019. 55(6):239.
2. Chaanine AH, LeJemtel T, Delafontaine P. Mitochondrial Pathobiology and Metabolic Remodeling in Progression to Overt Systolic Heart Failure. Journal of Clinical Medicine. 2020. 9. 3582.
3. Kong J, Xu Z. Massive mitochondrial degeneration in motor neurons triggers the onset of amyotrophic lateral sclerosis in mice expressing a mutant SOD1. J Neurosci. 1998. 18(9):3241-50.
4. Aliev G, Li Y, Palacios HH, Obrenovich ME, Bragin V, Bragin I, et al. Oxidative stress-induced mitochondrial damage as a hallmark for drug development in the context of the neurodegeneration, cardiovascular, and cerebrovascular diseases. In Systems Biology of Free Radicals and Antioxidants. Springer-Verlag Berlin Heidelberg 2012. p. 2083-2126.
5. Sasaki S, Iwata M. Mitochondrial Alterations in the Spinal Cord of Patients with Sporadic Amyotrophic Lateral Sclerosis. J Neuropathol Exp Neurol. 2007. 66(1):10-6.

Lines 172-173: Features of autophagic vesicles (“double or multiple membranes”) are not convincingly demonstrated in Figure 2c, inset.

**Replaced inset images with more recognizable or typical autophagic vesicles, and reworded the description to “… recognizable by their multivesicular myelin-like, or granular osmiophilic content…”, a description often used in literature**

1. Uchiyama Y, Shibata M., Koike M. et al. Autophagy–physiology and pathophysiology. Histochem Cell Biol. 2008. 129:407–420.
2. Eskelinen EL, Kallio K. Ultrastructure of the Macroautophagy Pathway in Mammalian Cells. In: Loos B, Wong E (eds). Imaging and Quantifying Neuronal Autophagy. Neuromethods. Vol 171. Humana, New York, NY. 2022. p 13-21
3. Koike M, Nakanishi H, Saftig P, Ezaki J, Isahara K,Ohsawa Y, et al. Cathepsin D Deficiency Induces Lysosomal Storage with Ceroid Lipofuscin in Mouse CNS Neurons. The Journal of Neuroscience. 2000. 20:6898-6906.
4. Eskelinen, EL. New Insights into the Mechanisms of Macroautophagy in Mammalian Cells. International Review of Cell and Molecular Biology. 2008. 266:207-247.

Figure 192-3: Especially in Figure 3b and c, it is not clear why structures seemingly representing electron-lucent vacuoles are assigned as dilated Golgi apparatus.

**The structures were labelled Golgi apparatus after consideration of several factors:**

- **Their perinuclear location.**
- **After thorough scrutiny of the cells, no continuity with the nuclear membrane could be found which might have indicated that the structures are smooth endoplasmic reticulum (ER).**
- **As the rough endoplasmic reticulum was largely unaffected, it is not expected that the smooth ER would show such a high degree of swelling, which convinced us that the “vacuoles” are not smooth ER.**
- **Even with the cisternae being dilated, in certain areas it can be seen that the “vacuoles” are stacked vesicular profiles.**
- **When compared to the Golgi apparatus of control cells, cells treated with lasalocid (which had less of an effect on the Golgi apparatus) or cells in which the Golgi apparatus structure could easily be discerned; the membrane is recognizable (electron density / thickness / folding) as those of the marked structures.**
- **Comparison to published images of dilated and swollen Golgi apparatus.**
- **And lastly; based on the knowledge of how ionophore exposure affect cells and what has been seen in other cell lines; swelling of the Golgi apparatus was expected and the finding in-line with that.**

1. Radulescu A, & Siddhanta A, Shields D. A Role for Clathrin in Reassembly of the Golgi Apparatus. Molecular biology of the cell. 2007. 18:94-105.
2. Berning L, Lenz T, Bergmann AK, Poschmann G, Brass HUC, Schlütermann D, et al. The Golgi stacking protein GRASP55 is targeted by the natural compound prodigiosin. Cell Commun Signal. 2023. 21: 275.
3. Podinovskaia M, Prescianotto-Baschong C, Buser DP, Spang A. A novel live-cell imaging assay reveals regulation of endosome maturation eLife. 2021. 10:e70982.
4. Zhang C, Rosenwald A, Willingham M, Skuntz , & Clark J, Kahn R. Expression of a dominant allele of human ARF1 inhibits membrane traffic in vivo. The Journal of cell biology. 1994. 124. 289-300.

Lines 214-215: It is not clear, what the bordered arrowheads are supposed to show in Figure 1e-1h. Line 156-157 indicate “filipodia-like and bleb-like surface structures” in Figure 1h that should be assigned.

**Higher magnification images added as insets to Figure 1h to show the blebs and filipodia, and the arrowhead shape was changed to clarify which corner is the one pointing to the relevant feature (changed in all the images). Additionally, a description “..filipodia- and bleb-like surface structures..” was added to the figure legend.**

Lines 227-228: Some of the arrowheads in Figure 3e clearly do not show surface indentations. In the results related to Figure 3, indentations are not covered at all as a feature of H9c2 exposed to salinomycin.

**The pockmarked appearance is mentioned in lines 187-188 , we added clarification to the text to highlight that the pocked marked appearance is due to the observed surface indentations. The arrowheads were repositioned to make sure they are pointing to an example of a surface indentation and the arrowhead shape was changed to clarify which corner is the one pointing to the relevant feature.**

Lines 253-254: At least Figure 6a seems not to show “myoblasts … filled with electron-lucent vacuoles…”.

**Figure 6b replaced to show the cell filled with vacuoles, and moved to Figure 6a position, and the original Figure 6a was moved to the Figure 6b position.**

Lines 270-271: When comparing Figure 7a with Figure 6a and b, it seems not to be justified to state that “Myoblasts exposed to salinomycin were less vacuolated than those exposed to monensin (Fig 7 a)…”.

**We exchanged figure 6a, which we hope better illustrate the difference between the two treatments. Additionally, the text was changed to clarify that less cells were extensively vacuolated, so it is not just the severity of the vacuolization it is also the number of cells affected.**

Line 314: The structure encircled in Figure 8e is supposed to show apoptotic bodies, that seem to have a quiet different structure as compared with the apoptotic bodies shown in Figure 2g, 3g, and 6g. Please comment.

**One difference between the images which creates the impression that the structure of these apoptotic bodies (ABs) are different is the magnification at which the ABs were imaged. Figures 2g, 3g, and 6g were imaged at roughly double the magnification of Figure 8e. When looking at another apoptotic L6 cell treated with lasalocid and imaged at a comparable magnification ()see below), the AB structure does look similar.**

**However, you are correct that there are some differences;**

1. **Firstly; although the cell is undergoing apoptosis and covered in ABs, the spindle cell shape is still recognizable in Figure 8e and,**
2. **Secondly when measuring the ABs in L6 cells treated with lasalocid, the ABs size mean is slightly smaller and the size range is also smaller, with a noticeable lack of larger ABs (>2** **µm)**

H9c2 + monensin	Size range: 1.6µm - 5µm	Mean size: 2.3µm
H9c2 + salinomycin	Size range: 0.7µm – 3.5µm	Mean size: 1.1µm
L6 + monensin	Size range: 1.0µm – 3.7µm	Mean size: 1.5µm
L6 + lasalocid	Size range: 0.6µm – 1.8µm	Mean size: 1.0µm

**The sizes of ABs in all of the exposed cells does still fall inside the general guide for ABs size (accepted to fall between 1µm - 5µm, however many cite 0.5µm - 5µm as a range and others have shown ABs as small as 100nm and as large as 10µm).**

**The differences in size and size range observed between the different ionophores and cell types during this study can be contributed to the fact that the size and number of ABs formed is dependent on the cell type, the pattern, mechanism and the course of cell death (see references below).**

**In conclusion we believe Figures 2g, 3g, 6g and 8e all represent ABs because:**

- **The ABs / particle sizes are within range of known AB size**
- **TEM images show typical ABs along with other morphological apoptotic features.**
- **The differences in ABs sizes was noticed and after review of the available literature, hypothesized to be due to the**
  - **different cell types which is seen to differ slightly in their response to different ionophores in this study.**
  - **The treatment of that particular sample with lasalocid which is known, and also shown in this study, to have the least effect on the myoblast, which could contribute to a different pattern, or slower progression of cell death which would affect AB size. This would also explain why the apoptotic cell spindle shape is still recognizable.**

**This observed differences and the explanations thereof is outside the scope of this manuscript**

1. Poon I, Parkes M, Lanzhou & Atkin-Smith G, Tixeira R, Gregory C, et al. (2019). Moving beyond size and phosphatidylserine exposure: evidence for a diversity of apoptotic cell-derived extracellular vesicles in vitro. Journal of Extracellular Vesicles. 2019. 8(1):1608786.
2. Crescitelli R, Lässer C, Szabó TG, Kittel A, Eldh M, Dianzani I, et al. Distinct RNA profiles in subpopulations of extracellular vesicles: apoptotic bodies, microvesicles and exosomes. Journal of Extracellular Vesicles. 2013. 2: 20677.
3. Xuebo X, Yueyang L, Zi-Chun H. Apoptosis and apoptotic body: disease message and therapeutic target potentials. Biosci Rep*.* 2019. 39(1):BSR20180992.
4. Yu F, Yifan H, Di W, Bingdong S, Yan J, Xuefeng H, et al. Apoptotic vesicles: emerging concepts and research progress in physiology and therapy. 2023. Life Medicine. 2023. 2(2).
5. Núñez R, Sancho-Martínez S, Novoa J, et al. Apoptotic volume decrease as a geometric determinant for cell dismantling into apoptotic bodies. Cell Death Differ 2010. 17:1665–1671.
6. López-Hernández FJ. Cell Surface Area to Volume Relationship During Apoptosis and Apoptotic Body Formation. Cell Physiol Biochem. 2021. 55(S1):161-170

Line 316: Golgi apparatus seems hardly to be visible in Figure 8c.

**We exchanged the image, and also added a higher magnification inset image to illustrate the Golgi apparatus.**

Lines 351-354: An effect of monenesin on the Golgi apparatus is neither demonstrated in Figure 2 for H9c2 myoblasts nor in Figure 6 for L6 myoblasts.

**The relevant passage has been reworded to highlight that the Golgi apparatus was not recognizable as stacked and organized cisternae after monensin exposure, and that vacuoles and vesicular profiles was all that was seen.**

The discussion, while in general improved and much more interesting, is now a little bit lengthy and not always very stringent.

**We rewrote the discussion section, focusing on the sections relating to published cell death mechanisms following ionophore exposure, condensing it and hopefully making it more coherent .**

**Minor points**

Line 151: should read: “in addition to” instead of “with some”.

**Changed “with some” to “in addition to”**

Line 170, line 362: Please delete “with”.

**Deleted “with**

Line 188-189: Please rephrase “…necrotic myoblasts were observed with little
to no membrane continuity, mostly only comprising cellular debris…”

**Rephrased “…necrotic myoblasts were observed with little to no membrane continuity, mostly only comprising cellular debris…” to “ necrotic myoblast, primarily consisting of cellular debris and exhibiting a nearly complete loss of membrane continuity, were observed”**

Lines 205 and 235: Please specify, what kind of surface structures are indicated by bordered arrowheads in Figures 4e-g.

**Specified in the text and figure legend that the bordered arrowheads indicate filipodia- and bleb-like surface structures.**

Line 214: Why are H9c2 myoblasts incubated in DMEM for 48 h termed as “negative control”?

**In all the experiments performed the cells were cultured and grown in Dulbecco’s Modified Eagle’s medium (DMEM). In the exposed groups monensin, salinomycin, or lasalocid was added, whereas in the control group no ionophore was added and the cells were further incubated in the medium it was cultured in. As the control cells were not exposed to the treatment, and not expected to produce any results, they are negative-controls and serves as a baseline for the ultrastructure of “normal / healthy” cells.**

Line 228: Should read “filipodia- and bleb-like surface structures” (line 199) instead of “surface structures”.

**Filipodia- and bleb-like added to the figure legend.**

Lines 220, 228, 300, and 314: Should read “encircled “.

**“Circled” changed to “encircled”**

Lines 306-307: Arrowheads are supposed to show indentations. Concerning Figure 7f, this statement is questionable.

**The arrowheads have been repositioned to attempt to show the indentations more clearly and the arrowhead shape was changed to clarify which corner is the one pointing to the relevant feature.**

Lines 307: Please specify, what kind of structures the bordered arrowheads are supposed to show.

**Specified that the bordered arrowheads indicate filipodia-, bleb-, or frill-like surface structures.**

Line 356: Please rephrase “The number of endoplasmic reticulum profiles also increased…”.

**Rephrased from “... number of endoplasmic reticulum profiles also increased...” to “…endoplasmic reticulum surface also increased...”**

Line 404. Please rephrase “vacuole-presenting cell death”.

**Rephrased ”…vacuole-presenting cell death...” to “…cell death modalities characterised by excessive vacuole formation…”**
